# Induction of Mitochondria-Mediated Apoptosis in Ca Ski Human Cervical Cancer Cells Triggered by Mollic Acid Arabinoside Isolated from *Leea indica*

**DOI:** 10.1155/2012/684740

**Published:** 2011-11-20

**Authors:** Yau Hsiung Wong, Habsah Abdul Kadir

**Affiliations:** Biomolecular Research Group, Biochemistry Program, Institute of Biological Sciences, Faculty of Science, University of Malaya, 50603 Kuala Lumpur, Malaysia

## Abstract

*Leea indica* is a medicinal plant traditionally used to treat cancer. Through bioassay-guided approach, we isolated mollic acid arabinoside (MAA), for the first time from *Leea indica*. Here, we present the apoptosis-inducing effect of MAA on Ca Ski cervical cancer cells. Based on DAPI staining, MAA-treated cells manifested nuclear shrinkage, condensation, and fragmentation. We further confirmed the fragmentation of DNA using TUNEL assay. During early apoptosis, MAA caused the perturbation of plasma membrane through externalization of PS, followed by the formation of apoptotic blebs. Prior to these events, MAA triggered rapid dissipation of the mitochondrial membrane potential. In the upstream, MAA increased the expression of Bax, decreased the expression of Bcl-2, and augmented the Bax/Bcl-2 ratio. These findings suggested that MAA induced mitochondrial-mediated apoptosis in Ca Ski cells and thus provide the scientific explanation for the traditional application of this herbal medicine in cancer treatment.

## 1. Introduction

Apoptosis is a well-defined process that is controlled by a sequence of regulated events that eventually resulted in demise of the cell. Apoptosis plays a fundamental role in maintaining normal tissue homeostasis by regulating the balance between cell proliferation and cell death [1]. Characteristically, it can be defined by several morphological and biochemical changes that affect the nucleus, plasma membrane, and mitochondria, including nuclear condensation (pyknosis), nuclear fragmentation (karyorrhexis), membrane blebbing, externalization of phosphatidylserine, and collapse of mitochondrial membrane potential (Δ*ψ*m) [2]. Induction of cell apoptosis is considered a useful approach in the treatment of cancer [3]. It has been reported that a variety of phytochemicals from natural products induced apoptosis in cancer cell lines. Indeed, many have been used as cancer chemopreventive agents [4]. In view of that, intensive efforts have been made to identify new bioactive compounds from natural products, through isolation of apoptosis inducing agents and elucidation of the apoptosis mechanisms. 

*Leea indica* (Burm. f.) Merrill (Leeaceae) is a medicinal plant widely distributed in tropical and subtropical places such as China, India, Malaysia, Thailand, and Indonesia. It has many local common names such as Bandicoot berry (English); Memali (Malay); huo tong shu (Chinese); katangbai (Thai); Hastipalash (India). The roots, leaves, and other parts of the plant are used traditionally for many medicinal purposes. The roots are used as decoction or drinks by the local for the treatment of colic, diarrhea, and dysentery [5, 6]. Extract of inflorescence and root is given to children in chest bulging to cure chest pain [7]. The leaves are consumed as herbal tea by the locals for general health. The juice of the leaves is taken in by woman as remedy during pregnancy and delivery, or for birth control [8], while the leaves decoction is used to treat obstetric diseases and body pain [9]. In addition, the leaves are taken orally to treat cold, headache, injury, or rheumatoid arthritis [10]. It is also an ingredient in the herbal preparation to treat cancers [11]. 

In our preliminary cytotoxicity screening, the crude ethanol extract and fractions (ethyl acetate, hexane, and water) of the leaves were found to inhibit the growth of Ca Ski cervical cancer cells, with the strongest growth-inhibitory effects shown by the *L. indica *ethyl acetate fraction (LIEAF) [12]. As part of our continuing studies, LIEAF was subjected to MTT assay-directed separation by using Ca Ski cells as a model. Through the bioassay-guided approach, mollic acid arabinoside (MAA), a cycloartane triterpenoid was isolated for the first time from LIEAF. MTT studies showed that MAA was cytotoxic to Ca Ski cells (IC_50_ of 19.21 *μ*M) after 72 h of incubation and much less cytotoxic to MRC5 normal cell (about 8-fold higher IC50) [13]. Recently, growing attention has been devoted to the studies on the apoptosis by cycloartane triterpenoids [14–18]. However, the apoptosis and the underlying mechanisms of MAA are still unknown. Therefore, in this study, we attempted to evaluate the apoptosis inducing effect and dissect the mechanism of apoptosis elicited by MAA in Ca Ski cervical cancer cells. 

## 2. Methods

### 2.1. Cell Culture

The human cervical epidermoid carcinoma cell line (Ca Ski, ATCC number CRL-1550) was used in the current study. It was purchased from American Type Culture Collection (ATCC, USA). The cells were cultured as monolayers in RPMI 1640 growth medium (Sigma), containing 10% (v/v) heat-inactivated fetal bovine serum (PAA), 100 *μ*g/mL penicillin/streptomycin (PAA), and 50 *μ*g/mL amphotericin B (PAA). The cells were maintained inside a CO_2_ incubator (Galaxy R, RS Biotech) at 37°C with moist atmosphere of 5% CO_2_ in air.

### 2.2. 4′,6-Diamidino-2-Phenylindole (DAPI) Staining

To monitor morphological alterations typical of apoptosis, we performed DAPI nuclear staining. After MAA treatment, the cells were harvested and washed with PBS, followed by fixation of cells in acetone at 4°C for 30 min. The fixed cells were washed with PBS and stained for 30 min with 1 *μ*g/mL DAPI in the room temperature. The stained cells were then examined for morphological signs of apoptosis under a Leica DM 2500 fluorescence microscope, and photographs were captured by a Leica DFC 310 FX camera.

### 2.3. Terminal Deoxynucleotidyl Transferase (TdT) dUTP Nick End Labeling (TUNEL) Assay

To detect DNA fragmentation, we used a TUNEL assay kit (Apo-BRDU, Sigma). The kit uses two dyes, FITC to label apoptotic DNA strand breaks (DSBs) and PI to stain total cellular DNA. DSBs were labeled with bromodeoxyuridine triphosphate nucleotides (BrdUTP) and detected by FITC-conjugated BrdUTP antibody. Briefly, after MAA treatment, 1 × 10^6^ cells were fixed for 15 min in ice-cold 1% (w/v) paraformaldehyde in PBS. The fixed cells were stored for at least 24 h in 70% (v/v) ethanol at −20°C. Following washing of the cells, they were incubated for 60 min with 50 *μ*L reaction buffer containing TdT enzyme and BrdUTP at 37°C. The incorporated BrdUTP was then labeled with 100 *μ*L FITC-conjugated BrdUTP antibody for 30 min, followed by addition of 400 *μ*L PI/RNase and further incubated for 30 min at room temperature. Then, the cells were subjected to analysis by FACS Calibur flow cytometry using Cell Quest software (BD). 

### 2.4. Analysis on Externalization of Phosphatidylserine (Annexin V/PI Staining)

Annexin V/PI staining was assessed to measure the externalization of PS during early apoptosis [19]. After MAA treatment, the washed cells were resuspended in Annexin V binding buffer (BD Biosciences) at the density of 1 ×  10^6^ cells/mL. 0.1 mL (1 × 10^5^ cells) of the cell suspension was then transferred to a 5 mL BD falcon round-bottom tube, where 5 *μ*L FITC-conjugated Annexin V (BD Biosciences) and 10 *μ*L PI (50 *μ*g/mL) were added. The cells were vortexed gently and incubated for 30 min at room temperature. An addition of 400 *μ*L binding buffer was added to the tubes before the cells were analyzed by FACS Calibur and Cell Quest software.

### 2.5. Apoptotic Bleb Analysis (ScFv/PI Staining)

The formation of membrane blebbing in apoptotic cells was measured using an apoptotic blebs assay kit (Cayman Chemical). The kit uses a recombinant protein, which was derived from single-chain variable fragments (scFv) of an antibody, to specifically recognize the autoantigens in the apoptotic blebs [20]. The recombinant protein is then detected using FITC-conjugated rabbit IgG. Briefly, after MAA treatment, 1 × 10^6^ cell suspensions were incubated for 30 min with scFv fusion protein at 4°C, followed by staining for 30 min with FITC-conjugated rabbit lgG at 4°C. After washing, the cells were stained for 10 min with PI at room temperature. The stained cells were washed twice and resuspended in 1 mL of PBS before analysis on FACS Calibur and Cell Quest software. 

### 2.6. Analysis on Mitochondrial Membrane Potential (JC-1 Staining)

The change in mitochondrial membrane potential (Δ*ψ*m) was measured using the 5,5′,6,6′-tetrachloro-1,1′,3,3′-tetraethyl benzimidazolyl carbocyanine iodide (JC-1) probe (stands for 1st J-aggregate-forming cationic dye). It is commonly used to detect mitochondrial depolarization that occurs in the early apoptosis [21, 22]. In healthy cells, JC-1 accumulates in the mitochondria as JC-1 aggregates (whose fluoresce is red) and also in the cytoplasm as JC-1 monomers (whose fluoresce is green). During early apoptosis, the Δ*ψ*m collapses. Consequently, JC-1 aggregates cannot accumulate within the mitochondria and dissipate into JC-1 monomers leading to loss of red fluorescences. Therefore, collapse of the Δ*ψ*m is signified by decrease in the ratio of red to green fluorescence [21]. Following MAA treatment, the cells were washed with PBS and incubated for 15 min with JC-1 at 37°C. After two times washings in PBS, the cells were subjected to two-color analysis by FACS Calibur and Cell Quest program. The red (JC-1 aggregate) and green (JC-1 monomer) fluorescence were detected at FL-2 and FL-1 channel, respectively.

### 2.7. Real-Time Quantitative PCR (Q-PCR)

To examine the role of Bcl-2 family members in MAA-induced apoptosis, we measured the gene expression of Bax and Bcl-2 using Q-PCR. 1 × 10^6^ cells were exposed to MAA for different exposure periods. After treatment exposure times, total RNA was isolated using RNAqueous-4PCR kit (Applied Biosystem) based on the manufacturer's protocol. Gene expression of Bcl-2 (B-cell CLL/lymphoma 2) and Bax (BCL2-associated X protein) was assessed by one-step SYBR green relative Q-PCR (RotorGene-6000 System, Qiagen) and normalized to PBGD (porphobilinogen deaminase) reference genes. The primer sequences were shown in Table 1. The reactions were carried out in a total volume of 25 *μ*L using the SensiMix One-Step Kit (Quantace). The PCR amplification for Bcl-2 was 45 cycles of 20 seconds at 95°C, 40 seconds at 42°C, and 10 seconds at 72°C. While for Bax it was 45 cycles of 20 seconds at 95°C, 40 seconds at 60°C, and 10 seconds at 72°C. The fluorescence threshold Ct values were calculated, and the ΔCt values were determined using the formula ΔCt = Ct_Bax  or  Bcl-2_−Ct_PBGD_. The ΔΔCt values were then calculated based on formula ΔΔCt = ΔCt treated−ΔCt untreated. The expression level of Bax or Bcl-2 in the treated cells was measured relatively to the level observed in the untreated cells and was quantitated using formula 2^−ΔΔCT^ [23]. 

### 2.8. Flow Cytometric Immunofluorescence Staining

The protein expression level of Bax and Bcl-2 was determined by immunofluorescence staining using flow cytometry. This method was based on Roussi et al. [24] with some modifications. Following MAA treatment, the cells were washed twice in PBS and then fixed and permeabilized using the Cytofix/Cytoperm kit (BD Biosciences). A total of 1 × 10^6^ cells were resuspended in 500 *μ*L of fixation/permeabilization solution and incubated for 20 min at 4°C. The cells were washed twice with Perm/Wash buffer and incubated for 15 min in 1 mL of Perm/Wash buffer. To detect Bax or Bcl-2, the fixed and permeabilized cells were incubated with 100 *μ*L of Perm/Wash buffer containing the antibodies. For Bcl-2 protein, the cells were stained directly for 30 min with 20 *μ*L FITC-conjugated mouse anti-human Bcl-2 monoclonal antibody (clone 124) or IgG1 isotype control (BD Biosciences) at 4°C. For indirect Bax staining, the cells were incubated for 30 min with either rabbit anti-human Bax polyclonal antibody or IgG1 isotype control (BD Biosciences) at 4°C. After washing, the cells were further incubated for 30 min with FITC-conjugated goat anti-rabbit F(ab′)2 polyclonal secondary antibody (Abcam) at 4°C. The cells were then washed with Perm/Wash buffer and analyzed using FACS Calibur and Cell Quest software. 

### 2.9. Statistical Analysis

The results are presented in means ± S.E. Statistical analysis of the results was performed using Student's *t*-test. *P* < 0.05 was considered to be significant difference. 

## 3. Results and Discussion 

### 3.1. MAA Elicited Nuclear Alterations Typical of Apoptotic Cell Death in Ca Ski Cells

**Figure 1:**
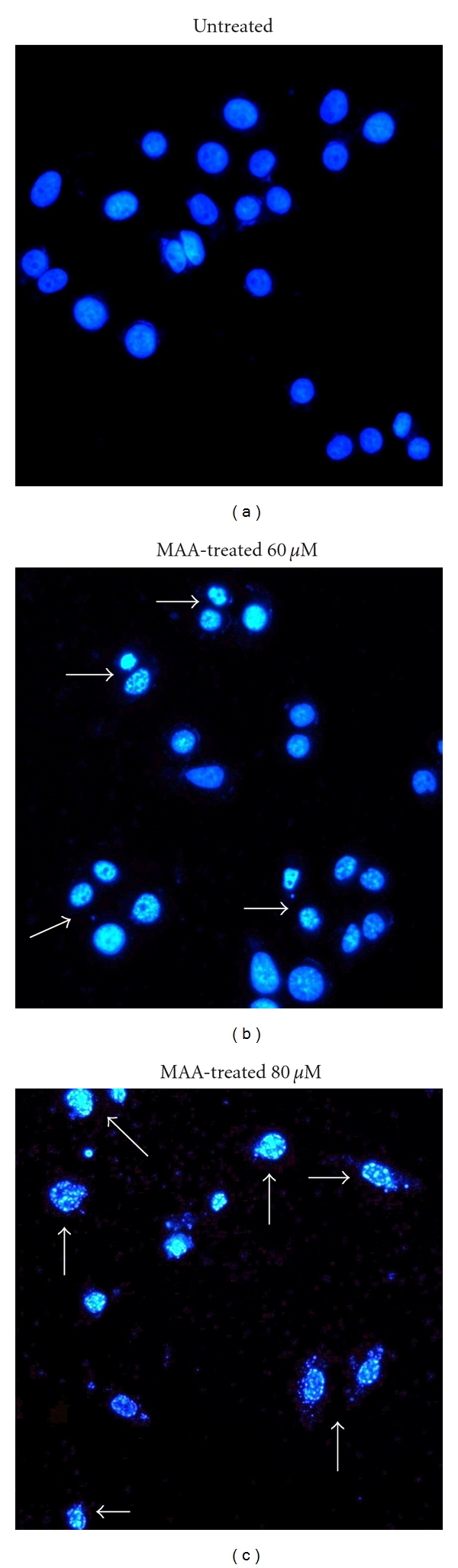
Effect of MAA on nuclear alterations in Ca Ski cells. Cells were incubated without or with 60 *μ*M and 80 *μ*M of MAA for 24 h. Cells were stained with DAPI and visualized by fluorescence microscopy. The arrows indicate nuclear shrinkage, condensation, or fragmentation with 100x magnification.

Apoptosis is initially characterized by morphological features, such as chromatin condensation, nuclear fragmentation, and membrane blebbing [2]. To gain an insight on the effect of MAA on nuclear alterations, cells were stained with DAPI. As shown in Figure 1, the cells underwent remarkable nuclear changes upon treatment. In the control untreated cells, the nuclei were intact, round, and uniformly stained. However, after exposed to MAA, the cells manifested two nuclear phenomena typical of apoptosis, namely pyknosis (nuclear shrinkage/condensation) and karyorrhexis (nuclear fragmentation). At 60 *μ*M of MAA, a number of cells exhibited nuclear shrinkage and chromatin condensation. At 80 *μ*M, the nuclear alterations were more apparent. The cells shrunk and lost its normal nuclear architecture. The chromatin was compacted into dense lumps and recognized as smaller intense spots. The nuclei were fragmented into apoptotic bodies (nuclear bodies). On the contrary, these aberrant nuclear alterations were not seen in the control cells. These observations showed that apoptosis occurred in Ca Ski cells after treatment of MAA. 

### 3.2. MAA Triggered DNA Fragmentation in Ca Ski Cells

The appearance of apoptotic nuclear alterations following MAA treatment led us to focus on another hallmark of apoptosis, DNA fragmentation. During late stage of apoptosis, endonucleases provoke the degradation of nuclear DNA, resulting in fragments of DNA strand breaks (DSBs) with exposed 3′-hydroxyl ends [25]. 

**Figure 2:**
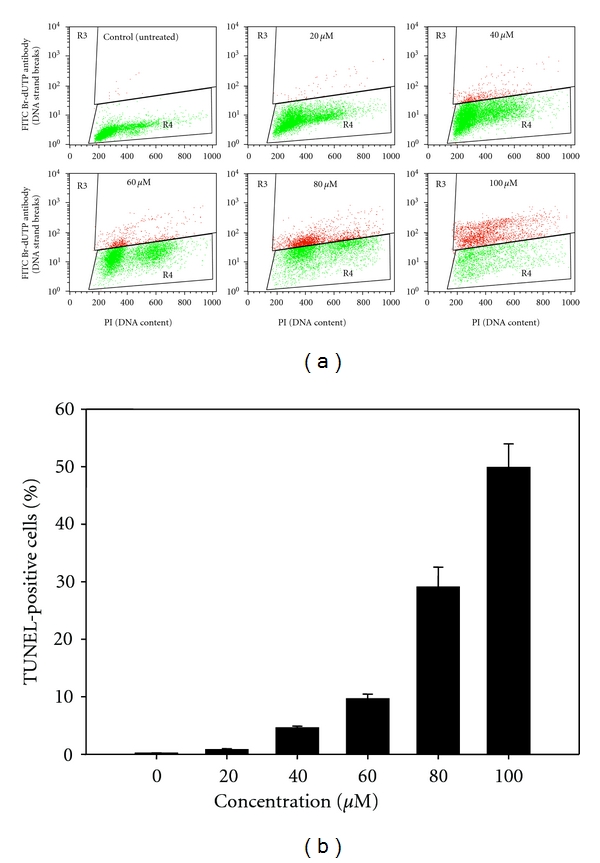
Effect of MAA on fragmentation of DNA in Ca Ski cells. Cells were exposed to different concentration of MAA for 24 h and proceeded to TUNEL assay as described in Section 2. (a) Representative dual parametric dot plots of PI (DNA content) on the *x*-axis and FITC Br-dUTP antibody (DNA strand breaks) on the *y*-axis. The gated R_3_ regions (red) indicate TUNEL-positive cells. (b) Bar chart shows the percentage of TUNEL-positive cells. Values are mean ± S.E. from three experiments.

To detect the generation of DSBs in Ca Ski cells, we performed TUNEL assay using the Apo-BRDU kit (Sigma). In this assay, TdT enzyme catalyzed the addition of BrdUTP to the exposed 3′-hydroxyl ends of DSBs. The incorporated BrdUTP was then detected with FITC-conjugated BrdUTP antibody. Simultaneously, the DNA content was measured to correlate the apoptosis with cell cycle [26]. As illustrated in Figure 2(a), in the absence of MAA, the majority of the untreated cells showed negative TUNEL staining. After MAA treatment, a number of cells acquired TUNEL staining, indicating the occurrence of DNA fragmentation. The effect of DNA fragmentation was found intensified in a dose-dependent way (Figure 2(b)). The effect seemed to occur at the S phase of cell cycle. When treated with 100 *μ*M of MAA, nearly 50% of the cells underwent DNA fragmentation and was found to appear in all phases of the cell cycle.

The increase in TUNEL positivity was in parallel with the appearance of cells with apoptotic nuclear alterations. This further confirmed the DAPI morphological-based results. Thus, we can postulate that DNA fragmentation, particularly breakage of DNA strands, might contribute to the aberrant nuclear alterations. Collectively, the observations from DAPI and TUNEL assays showed that the cells underwent apoptotic DNA damage after treatment with MAA. 

### 3.3. MAA Provoked the Externalization of PS in Ca Ski Cells

**Figure 3:**
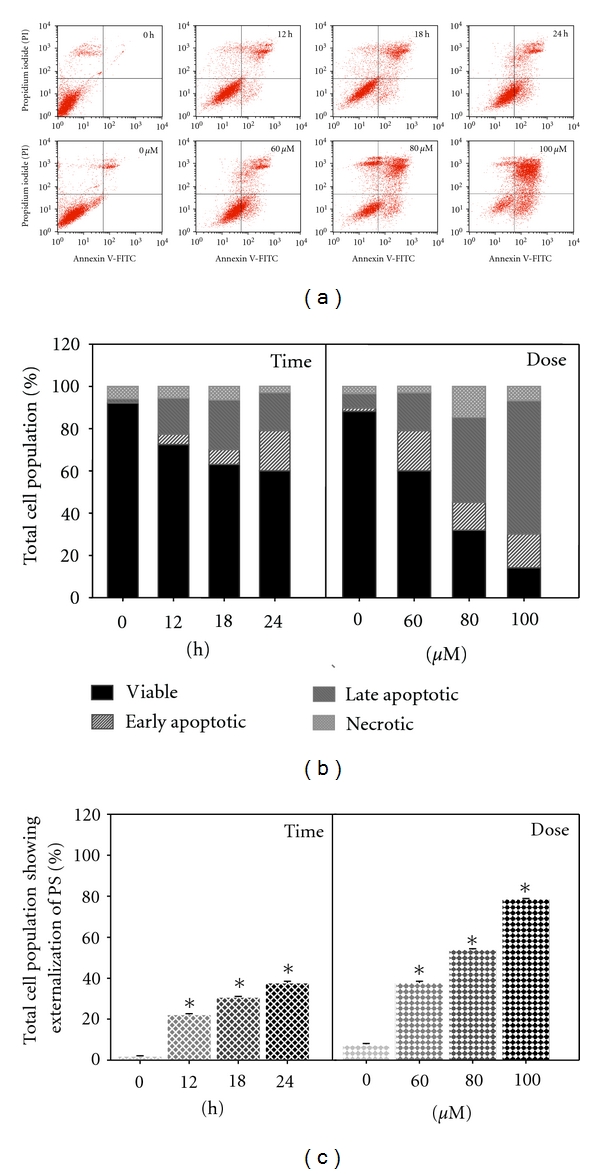
Effect of MAA on externalization of PS in Ca Ski cells. Cells were treated with 60 *μ*M of MAA for different time points. At 24 h of treatment, cells were treated with increasing concentration of MAA. At each time point, cells were collected and double stained with Annexin V-FITC and PI as described in Section 2. (a) Representative dual parametric dot plots of PI fluorescence (*y*-axis) versus Annexin V-FITC fluorescence (*x*-axis). (b) Bar charts showing the proportion of viable, early apoptotic, late apoptotic, and necrotic cells. (c) Bar charts show the percentage of Annexin V-positive cells (Annexin V+/PI− and Annexin V+/PI+). Values are mean ± S.E. of three experiments. Asterisks indicate a significant difference between untreated and treated cells (*P < 0.05).

The aforementioned results demonstrated the typical nuclear alterations and damages that occurred during late apoptosis. Subsequently, this prompted us to investigate the early event of apoptosis. Numerous studies have reported that advanced DNA fragmentation is preceded by alteration in the plasma membrane, such as PS externalization. During early apoptosis, the plasma membrane asymmetry is lost due to the externalization of PS [19]. Hence, MAA-treated cells were double stained with Annexin V-FITC/PI and analyzed by flow cytometry. Since 60 *μ*M of MAA elicited apoptotic responses in the DAPI and TUNEL assay, this concentration was selected for time-course measurement of the apoptotic process. According to Figures 3(a) and 3(b), untreated cells showed low or negative staining with both Annexin V and PI (Annexin V−/PI−), indicating viable cells. The early apoptotic cells (Annexin V+/PI−) began to appear as earlier as 12 h of treatment and were found increased in a time-dependent manner till 24 h, while the late apoptotic cells (Annexin V+/PI+) also increased after 12–24 h of exposure to MAA. Notably, a considerable accumulation of early and late apoptotic cells (approximately 20% for each) with less than 3% necrotic cells (Annexin V−PI+) was visible at 24 h. Consistent with the progression of apoptosis, the late apoptotic cells become dominant at later times, because we observed gradual diminution of the early apoptotic cells and increment of the late apoptotic cells after 24 h (data not shown). We also challenge the cells with increasing doses of MAA at 24 h of treatment. The results clearly showed the progression from early to late apoptosis, indicating by the dose-dependent accumulation of the late apoptotic cells. Overall, the combined early and late apoptotic cells (Annexin V positive) were elevated in a time- and dose-dependent fashion (Figure 3(c)). It is important to note that, at every treatment dose and exposure time, the necrotic cell population was small (below 15%). This indicated that apoptosis is the preferential cell death induced by MAA in Ca Ski cells. 

### 3.4. MAA Induced the Formation of Apoptotic Blebs in Ca Ski Cells

**Figure 4:**
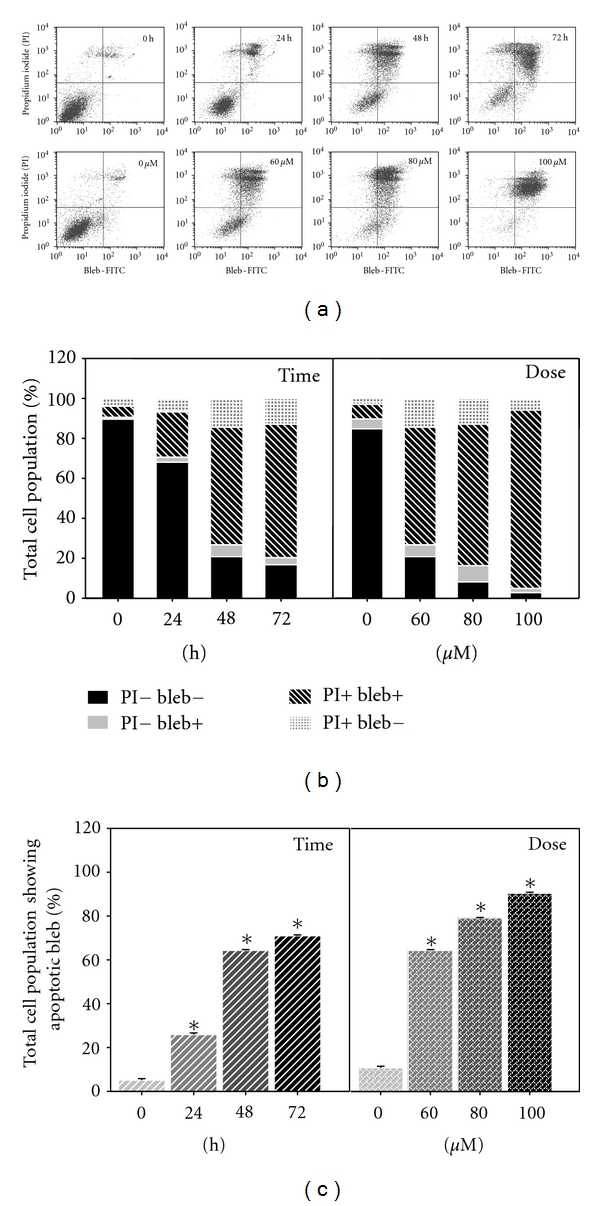
Effect of MAA on formation of apoptotic bleb in Ca Ski cells. Cells were treated with 60 *μ*M of MAA for different time points. At 48 h of treatment, cells were treated with increasing concentration of MAA. After treatment, the cells were processed for apoptotic blebs staining and analyzed by flow cytometry as described in Section 2. (a) Representative dual parametric dot plots of PI fluorescence (*y*-axis) versus bleb-FITC fluorescence (*x*-axis). Bleb-FITC represents the binding of apoptotic blebs by scFv protein which was labeled with FITC-conjugated rabbit lgG. (b) Bar charts show the proportion of total stained cells based on flow cytometry analysis. (c) Bar charts show the percentage of total cell population with apoptotic blebs (PI− bleb+ and PI+ bleb+). Values are mean ± S.E. of three experiments. Asterisks indicate a significant difference between untreated and treated cells (*P < 0.05).

Beside externalization of PS, another dramatic change to the plasma membrane during apoptosis is the surface protrusion known as blebs [2]. Apoptotic cells package autoantigens (such as nuclear fragments and DNA) in vesicles and distributed them to the membrane surface as blebs [20]. To further explore this apoptotic phenotype, we used a commercial apoptotic bleb assay kit. The kit is based on the binding of scFv fusion protein to the antoantigens in apoptotic blebs, followed by detection with FITC-conjugated antibody [20]. As shown in Figures 4(a) and 4(b), the majority of untreated cells were not stained with both the bleb-FITC and PI. However, after treatment with MAA, they were progressively stained with the bleb-FITC. We noted that most of the treated cells were double stained with both bleb-FITC and PI (PI+ bleb+). This finding was in accordance with other reports which showed that scFv preferentially binds to cells in the later stage of apoptosis [27, 28]. This was also consistent with the fact that the surface blebs of apoptotic cells contained nuclear antigens such as DNA [20]. As shown in Figure 4(c), when compared to untreated cells, the treated cells showed significant increase of apoptotic blebs in a time- and dose-dependent manner. Similar to Annexin V/PI staining, the primary necrotic cell population (PI+ bleb−) in every treatment dose and exposure time was less than 15%. This again confirmed the proapoptotic nature of MAA in Ca Ski cells. 

Based on our time course experiments, externalization of PS (12 h) took place before the formation of apoptotic blebs (24 h). This was in agreement with other scholars finding which showed that Annexin V binding precedes scFv binding [20]. Taken together, the apoptosis process triggered by MAA in Ca Ski cells involved the aberrant changes to the cell membrane. During early hour (12 h) following MAA treatment, the membrane asymmetry was lost as PS was translocated from the inner to outer membrane leaflet. Longer treatments (24–72 h) of MAA evoked the appearance of blebs. 

### 3.5. MAA Dissipated the Mitochondrial Membrane Potential (Δ*ψ*m) in Ca Ski Cells

Besides PS externalization, dissipation of Δ*ψ*m has also been reported to be an early apoptosis event in many different systems [29]. It is a rapid and irreversible event preceding other manifestation of apoptosis process, such as PS externalization, nuclear DNA cleavage, and chromatin condensation [30, 31]. 

**Figure 5:**
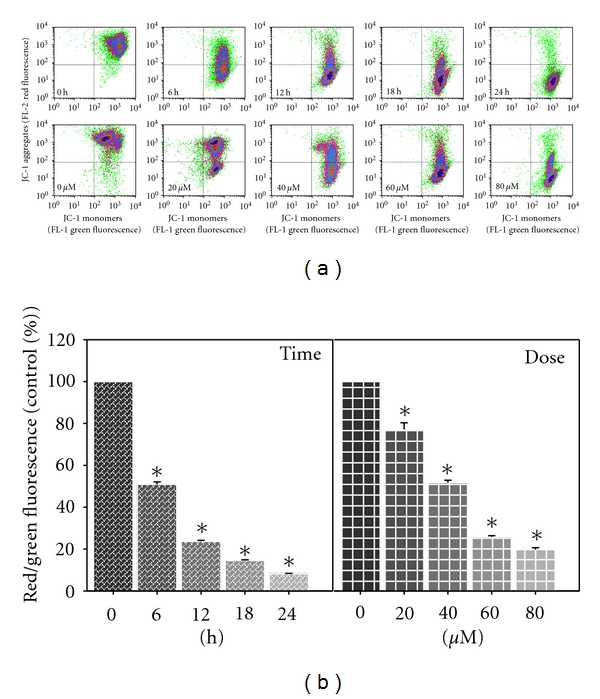
Effect of MAA on mitochondrial membrane potential (Δ*ψ*m) in Ca Ski cells. Cells were treated with 60 *μ*M of MAA for different time points. At 12 h of treatment, cells were treated with different concentration of MAA. After treatment, the cells were stained with JC-1, and the change in Δ*ψ*m was analyzed by flow cytometry as described in Section 2. (a) Representative dot plots of JC-1 aggregates (FL-2 red fluorescence) versus JC-1 monomers (FL-1 green fluorescence). (b) Bar charts showing the ratio of red/green fluorescence, expressed as percentage of control, indicate the ratio of high/low Δ*ψ*m. Values are mean ± S.E. of three experiments. Asterisks indicate a significant difference between untreated and treated cells (*P < 0.05).

To signal the loss of Δ*ψ*m, JC-1 probe was applied. As shown in Figure 5(a), the majority of the untreated cells were identified in the upper right quadrant (where both JC-1 aggregates and monomers were detected). This corresponded to mitochondria with a polarized Δ*ψ*m. However, after treatment of MAA, the cells showed progressive loss of JC-1 aggregates (decreased red fluorescence in the FL-2 channel). Consequently, the treated cells had lower red fluorescence than the untreated cells. This corresponded to mitochondria with a depolarized Δ*ψ*m. As indicated by JC-1 red/green fluorescence ratio (Figure 5(b)), the loss of Δ*ψ*m occurred in a time- and dose-dependent manner. According to the time course study, MAA induced a rapid (6 h) collapse of Δ*ψ*m, which preceded PS externalization (12 h). These data indicated that the MAA-induced apoptosis in Ca Ski cells involved mitochondria dysfunction associated with dissipation of the Δ*ψ*m.

### 3.6. MAA Increased the Expression of Bax, Decreased the Expression of Bcl-2, and Augmented the Ratio of Bax/Bcl-2

The members of the Bcl-2 family play a pivotal role in the regulation of the mitochondrial apoptotic pathway [32]. Among the members, the antiapoptotic Bcl-2 inhibits apoptosis, whereas the proapoptotic Bax counterbalances the Bcl-2 effect and stimulates apoptosis [33]. Other studies have implicated that Bcl-2 maintained the mitochondrial integrity, while Bax destroyed the mitochondrial integrity and caused loss of Δ*ψ*m [34]. Consequently, the ratio between Bcl-2 and Bax determines the susceptibility to apoptosis and thus dictates the fate of life and death of a cell [35, 36].

**Figure 6:**
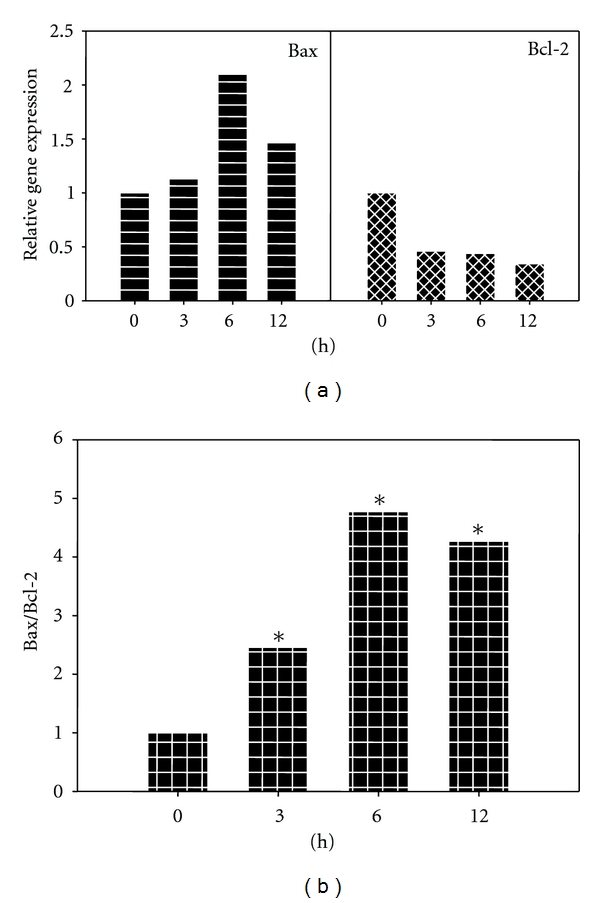
Effect of MAA on gene expression level of Bax and Bcl-2 in Ca Ski cells. Cells were treated with 60 *μ*M MAA for different times. After designated exposure times, cells were harvested and the total RNA was extracted. The RNA was subjected to Q-PCR analysis as described in Section 2. (a) The relative gene expression level of Bax and Bcl-2 was compared to control, which was normalized against PBGD expression using the formula 2^−ΔΔCT^. (b) Bar charts show the ratio of Bax/Bcl-2, analyzed from the Q-PCR results. Values are mean ± S.E. of three experiments. Asterisks indicate a significant difference between untreated and treated cells (*P < 0.05).

It has been known that Δ*ψ*m plays a pivotal role in mitochondria-mediated apoptosis [31]. Since MAA showed the ability to interfere with the Δ*ψ*m, we raised the possibility that Bax and Bcl-2 are involved in the MAA-induced apoptosis. Therefore, we investigated the gene expression level of Bax and Bcl-2 using Q-PCR. As depicted in Figure 6(a), Bax expression increased after 3 h of treatment, reached its peak at 6 h (about 2-fold higher compared to untreated), and reduced after that at 12 h. Since apoptosis is regulated by many genes, one possible explanation is that the proapoptotic activity of Bax could be negated by increased level of other proapoptotic regulated genes which are not investigated in this study. Despite the reduction in Bax expression at 12 h, Bcl-2 expression was clearly suppressed as it was found decreased 2-fold at 3 h and remained lower during the treatment hours. The increase in Bax and decrease in Bcl-2 expression significantly elevated the Bax/Bcl-2 expression ratio (Figure 6(b)) and thus sensitized the cells to apoptotic stimuli. Taken together, these results showed that MAA was able to induce apoptosis by altering the regulation of apoptotic genes, particularly through the upregulation of Bax and downregulation of Bcl-2. 

**Figure 7:**
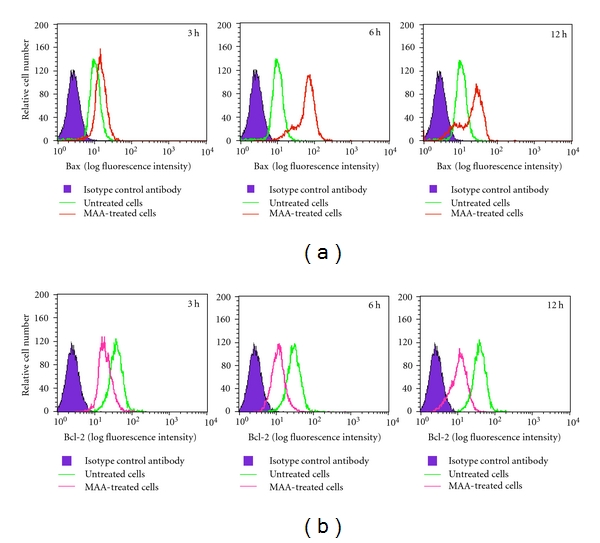
Effect of MAA on protein expression level of Bax and Bcl-2 in Ca Ski cells. After treated with 60 *μ*M MAA for different times, the cells were harvested and the intracellular levels of Bax and Bcl-2 were determined as described in Section 2. Representative overlay of histograms shows Bax- and Bcl-2-associated immunofluorescence.

Next, we applied flow cytometric immunofluorescence staining to confirm the expression of Bax and Bcl-2 in individual cells. As seen in Figure 7, when compared to the untreated cells, the MAA-treated cells profiles shifted to the right in the Bax histograms and to the left in the Bcl-2 histogram. Respectively, these reflected an increase and a decrease of Bax- and Bcl-2-associated immunofluorescence. This was in consistent with the Q-PCR results, in which Bax expression was reduced and Bcl-2 expression was augmented upon treatment with MAA. Altogether, the results support the idea that the MAA-induced apoptosis in Ca Ski cells was mediated by upregulation of Bax and downregulation of Bcl-2.

In the present study, we found that MAA promoted upregulation of Bax and downregulation of Bcl-2 as early as 3 h after the treatment. It is plausible that this may have been contributed to the disappearance of Δ*ψ*m that occurred later. Downstream of Bax/Bcl-2, two early apoptotic phenomena were observed, namely, PS externalization and Δ*ψ*m dissipation. It is interesting to note that the decrease in Δ*ψ*m (6 h) preceded the externalization of PS (12 h). These early apoptotic phenomena occurred before cells manifested other apoptotic phenotypes, such as nuclear condensation, DNA fragmentation, and membrane blebbing measured by DAPI, TUNEL, and apoptotic bleb assay. Based on our results, it appeared that Bax and Bcl-2 played a key role in execution of MAA-induced apoptosis in Ca Ski cells. We postulated that the marked increase in Bax/Bcl-2 ratio might be switching on the mitochondria-mediated apoptotic cascades. The cascade is further amplified by substantial disruption of Δ*ψ*m. This triggers activation of downstream events such as PS externalization, membrane blebbing, nuclear condensation, and DNA fragmentation that eventually culminates in cellular apoptosis. Collectively, our results suggested that the apoptosis induced by MAA in Ca Ski cells was mediated via the mitochondrial intrinsic pathway since MAA targeted the mitochondria and disturbed the mitochondrial functions, as can be seen from the disruption of Δ*ψ*m and altered expression of the mitochondrial-related proapoptotic and antiapoptotic molecules. Nonetheless, its possible role in the extrinsic pathway still needs to be explored in the future studies.

## 4. Conclusion

In conclusion, these findings unambiguously demonstrated that MAA isolated from *L. indica* induced mitochondrial-mediated apoptotic cell death in Ca Ski cells. Our findings may also pave the way for future development as cancer chemopreventive agents.

## Figures and Tables

**Table 1:** The primer sequence for Bcl-2, Bax, and PBGD.

Target genes	Function	Sequence
Bcl-2	Forward primer	5′-TTGGCCCCCGTTGCTT-3′
	Reverse primer	5′-CGGTTATCGTACCCCGTTCTC-3′

Bax	Forward primer	5′-GTCGCCCTTTTCTACTTTGCCAG-3′
	Reverse primer	5′-TCCAGCCCAACAGCCGCTCC-3′

PBGD	Forward primer	5′-ACCATCGGAGCCATCTGCAAG-3′
	Reverse primer	5′-CCCACCACACTCTTCTCTGGCA-3′
